# Oxidative DNA Damage Modulates DNA Methylation Pattern in Human Breast Cancer 1 (BRCA1) Gene via the Crosstalk between DNA Polymerase β and a *de novo* DNA Methyltransferase

**DOI:** 10.3390/cells9010225

**Published:** 2020-01-16

**Authors:** Zhongliang Jiang, Yanhao Lai, Jill M. Beaver, Pawlos S. Tsegay, Ming-Lang Zhao, Julie K. Horton, Marco Zamora, Hayley L. Rein, Frank Miralles, Mohammad Shaver, Joshua D. Hutcheson, Irina Agoulnik, Samuel H. Wilson, Yuan Liu

**Affiliations:** 1 Biochemistry Ph.D. Program, Florida International University, Miami, FL 33199, USA; zjian005@fiu.edu (Z.J.); jbeav004@fiu.edu (J.M.B.); ptseg001@fiu.edu (P.S.T.); 2 Department of Chemistry and Biochemistry, Florida International University, Miami, FL 33199, USA; yalai@fiu.edu (Y.L.); mzamo036@fiu.edu (M.Z.); hayley4@mail.usf.edu (H.L.R.); fmira020@fiu.edu (F.M.); 3 Genome Integrity and Structural Biology Laboratory, National Institute of Environmental Health Sciences, National Institutes of Health, Research Triangle Park, NC 27709, USA; zhaom2@niehs.nih.gov (M.-L.Z.); horton1@niehs.nih.gov (J.K.H.); wilson5@niehs.nih.gov (S.H.W.); 4 Department of Biomedical Engineering, Florida International University, Miami, FL 33199, USA; mshav001@fiu.edu (M.S.); jhutches@fiu.edu (J.D.H.); 5 Biomolecular Sciences Institute, Florida International University, Miami, FL 33199, USA; iagoulni@fiu.edu; 6 Department of Human and Molecular Genetics, Florida International University, Miami, FL 33199, USA

**Keywords:** oxidative DNA damage, DNA polymerase β, DNA methyltransferase 3b, BRCA1, base excision repair, DNA methylation, *de novo* DNA methylation

## Abstract

DNA damage and base excision repair (BER) are actively involved in the modulation of DNA methylation and demethylation. However, the underlying molecular mechanisms remain unclear. In this study, we seek to understand the mechanisms by exploring the effects of oxidative DNA damage on the DNA methylation pattern of the tumor suppressor breast cancer 1 (BRCA1) gene in the human embryonic kidney (HEK) HEK293H cells. We found that oxidative DNA damage simultaneously induced DNA demethylation and generation of new methylation sites at the CpGs located at the promoter and transcribed regions of the gene ranging from −189 to +27 in human cells. We demonstrated that DNA damage-induced demethylation was mediated by nucleotide misincorporation by DNA polymerase β (pol β). Surprisingly, we found that the generation of new DNA methylation sites was mediated by coordination between pol β and the *de novo* DNA methyltransferase, DNA methyltransferase 3b (DNMT3b), through the interaction between the two enzymes in the promoter and encoding regions of the BRCA1 gene. Our study provides the first evidence that oxidative DNA damage can cause dynamic changes in DNA methylation in the BRCA1 gene through the crosstalk between BER and *de novo* DNA methylation.

## 1. Introduction

DNA methylation, i.e., methylation at the 5-position of cytosine (5mC), plays an important role in gene expression, genomic imprinting, X-chromosome inactivation, embryonic development, and silencing of retrovirus. It is associated with genomic instability, aging, cancer, and neurodegeneration [1,2,3,4]. In the human genome, 60–80% of cytosines in CpG dinucleotides are methylated [3]. Unmethylated and methylated CpGs distribute at the promoter regions of genes to form a unique pattern in a gene- and tissue-specific manner. A normal DNA methylation pattern at a specific gene promoter is maintained upon the balance between DNA methylation and demethylation. DNA methylation is maintained by DNA methyltransferases (DNMTs) [5], whereas DNA demethylation can be accomplished by passive and active DNA demethylation pathways [6,7,8] through the inhibition of the activities of DNA methyltransferases and the DNA base excision repair (BER) pathway [9,10,11,12,13]. Active DNA demethylation is initiated by the conversion of a 5mC into a modified DNA base that is then removed by a DNA glycosylase. Subsequently, the 5mC is replaced by a C through BER, leading to DNA demethylation [9]. A recent study has shown that DNMT1 and DNMT3 can also result in DNA demethylation by producing the DNA base lesion 3-methylcytosine (3mC) [14], suggesting an alternative pathway of active DNA demethylation through the “off-target effect” of DNMTs. 

Dynamic changes in the patterns of DNA methylation regulate gene expression and sustain normal cellular function in a gene-, cell-, and tissue-specific manner. They can be readily modified by endogenous and exogenous factors. These include modulation of enzymatic activities for the addition and removal of a methyl group at the 5-position of cytosine; cellular abundance of the cofactor of DNA methyltransferases, S-adenosylmethionine (SAM); and DNA base modifications such as base lesions. Cytosines and guanines in CpG dinucleotides can be readily alkylated and oxidized [15]. These base lesions may inhibit the activity of DNMTs, preventing methylation of cytosine. Moreover, the deamination of 5mCs can directly result in a T/G mismatch, leading to the loss of 5mCs. All these can result in passive demethylation of a 5mC, thereby disrupting a normal DNA methylation pattern [10]. Thus, DNA base damage, BER, and DNMTs in cells may interplay to regulate the dynamics of DNA methylation in genes such as tumor suppressors. 

The tumor suppressor breast cancer 1 DNA repair-associated gene (BRCA1) was initially identified as a tumor suppressor of breast and ovarian cancer [16]. However, the protein is expressed ubiquitously in all organs and tissues with lymph nodes and skin having the highest level, followed by kidney and brain (V18.1 proteinatlas.org, The Human Protein Atlas). BRCA1 protein can directly participate in double-strand DNA (dsDNA) break repair [16]. It is also involved in chromatin remodeling and gene transcription, regulation of cell cycle checkpoint, centrosome regulation, apoptosis, and mitophagy [16]. Deficiency of BRCA1 function is associated with breast and ovarian cancer [17,18,19] as well as other types of cancer [20,21,22]. A recent study also demonstrates a significant increase of the BRCA1 gene expression in the brain of Alzheimer’s disease (AD) patients, suggesting an important role of BRCA1 in combating oxidative DNA damage that occurs in AD patients’ brains [23]. Thus, maintenance of a normal level and integrity of BRCA1 protein in cells is crucial for the sustainment of normal cellular function and the prevention of cancer and neurodegeneration. It is well known that mutations of BRCA1 proteins are responsible for familial breast cancer, whereas a low level of BRCA1 gene expression is associated with sporadic breast cancer [24,25,26]. Since more than 90% of breast cancer cases do not have a mutation in the encoding sequence of BRCA1 protein [26,27], it is proposed that downregulation of the BRCA1 gene expression may be one of the major causes of nonfamilial sporadic breast cancer [18,25,27,28,29]. This is supported by the fact that DNA hypermethylation at the promoter regions of the BRCA1 gene is associated with its downregulation in nonfamilial breast cancer [18,26] and that inhibition of DNMT activities can prevent the development of breast cancer [30]. This further suggests that the aberrant DNA methylation pattern underlies the deregulation of BRCA1 gene expression in human cancer. Since CpGs on the BRCA1 promoter and transcribed regions are the hotspots of DNA base lesions, it is possible that DNA base lesions and their repair can readily modulate the DNA methylation pattern of the tumor suppressor gene, leading to its expression deregulation. However, it remains unknown whether and how DNA base damage can modify the DNA methylation pattern at the BRCA1 gene. Understanding the underlying mechanisms is critically important for further understanding the interplay among the epigenetics of BRCA1, DNA damage, and repair and its role in the etiology of cancer and neurodegenerative diseases such as AD.

Among all types of DNA damage, DNA base damage is the most common form of DNA damage in mammalian cells. They include 8-oxoguanine (8-oxoG) [31], uracil, abasic sites, and T/G mismatch [14] that can occur at CpG dinucleotides and can directly inhibit the activities of DNMTs [14], leading to passive DNA demethylation. Also, in a scenario where BER simultaneously mediates active DNA demethylation and repairs DNA base lesions at CpGs, it can result in mutations through nucleotide misinsertions by DNA polymerase β (pol β) [32,33], leading to passive DNA demethylation. It is conceivable that DNA base lesions can inhibit DNMTs and cause base loss and nucleotide misincorporation during BER, thereby modulating the DNA methylation patterns of genes. We further hypothesize that oxidative DNA damage induces base lesions at the CpG dinucleotides in the promoter and transcribed regions of the BRCA1 gene to modulate the DNA methylation pattern of the gene through base loss and nucleotide misinsertion during BER. To test this, we examined the effects of oxidative DNA damage on the DNA methylation pattern of the human BRCA1 gene using human embryonic kidney 293H (HEK293H) cells as a model. This allows us to determine how DNA damage can modulate the DNA methylation pattern in the BRCA1 gene in normal cells. We found that oxidative DNA damage altered the DNA methylation pattern of the BRCA1 gene through the loss of 5mCs and point mutations at the CpG dinucleotides of the BRCA1 gene through BER. Surprisingly, we also found that oxidative DNA damage induced new DNA methylation sites at the BRCA1 gene. We further demonstrated that this was mediated by the interaction between pol β and the *de novo* DNA methyltransferase, DNMT3b, at the promoter and transcribed regions of the BRCA1 gene, indicating the cooperation between pol β and DNMT3b in *de novo* DNA methylation during BER. Our study provides the first evidence of demonstrating a crucial role of pol β and its crosstalk with a *de novo* methyltransferase in mediating the dynamic changes of DNA methylation patterns induced by oxidative DNA damage at the BRCA1 gene. 

## 2. Materials and Methods

### 2.1. Cell Lines and Materials

HEK293H cells were purchased from Life Technologies (Carlsbad, CA, USA). Fetal bovine serum (FBS) and Dulbecco’s Modified Eagle’s Medium (DMEM) high glucose cell culture medium was purchased from Thermo Fisher Scientific (Waltham, MA, USA). All other standard chemical reagents were purchased from Thermo Fisher Scientific (Waltham, MA, USA) and Sigma-Aldrich (St. Louis, MO, USA).

The genomic DNA isolation kit was purchased from Promega (Madison, WI, USA). The Lightning Bisulfite Conversion kit was purchased from ZYMO Research (Irvine, CA, USA). The Dream Taq polymerase master mix and the Original TA Cloning kit were purchased from Invitrogen (Carlsbad, CA, USA). The Rapid DNA Ligation kit was purchased from Thermo Scientific (Waltham, MA, USA), and the BigDye kit for sequencing was purchased from Applied Biosystems (Foster City, CA, USA).

The DNA oligonucleotides containing a 5mC and/or an 8-oxoG were synthesized by Midland Certified Reagent Company Inc. (Midland, TX, USA). All other oligonucleotides were synthesized by Integrated DNA Technologies (IDT, Coralville, IA, USA). Deoxynucleoside 5′-triphosphates (dNTPs) were purchased from Sigma-Aldrich (St. Louis, MO, USA). Radionucleotides, γ-^32^P-ATP (6000 µCi/mmol) was purchased from Perkin Elmer (Waltham, MA, USA). Micro Bio-Spin 6 chromatography columns were from Bio-Rad (Hercules, CA, USA). 

Human pol β was expressed and purified as described previously [34]. Polyclonal anti-pol β primary antibody (ab26343, Abcam, Cambridge, MA, USA), monoclonal anti-pol β primary antibody (ab175197, Abcam, Cambridge, MA, USA), polyclonal anti-DNMT3b primary antibody (ab2851, Abcam, Cambridge, MA, USA), polyclonal anti-DNMT1 primary antibody (ab19905, Abcam, Cambridge, MA, USA), and monoclonal anti-DNMT1 primary antibody (ab13537, Abcam, Cambridge, MA, USA) were purchased from Abcam (Cambridge, MA, USA). Information on the antibodies for specific experiments is provided in Supplementary Table S1. Pierce protease inhibitor tablets and protein A agarose beads were purchased from Thermo Fisher Scientific (Waltham, MA, USA). 

### 2.2. Oligonucleotide Substrates

Oligonucleotide substrates containing a 76-nt template strand with an 8-oxoG at the 38th nucleotide counted from the 5′-end were designed to mimic an oxidative base lesion generated in the genome. Oligonucleotide substrates containing a 76-nt template strand with a 5-mC and an 8-oxoG at the 37th and 38th positions counted from the 5′-end were designed to test the influence of 5-mC in the bypass of 8-oxoG. Substrates mimicking the intermediates with a 1-nt gap without or with a 5′-phosphorylated tetrahydrofuran (THF, an analog of a sugar residue) opposite a template 8-oxoG generated during BER were constructed by annealing the 38-nt upstream primer and a 37-nt downstream primer with a 5′-phosphate or 5′-phosphorylated THF residue with the template strand containing a 5mC and/or an 8-oxoG at a molar ratio of 1:2:2. Substrates were ^32^P-labeled at the 5′-end of the upstream primers for measuring the activities of different enzymes. The sequences of the oligonucleotide substrates are listed in Supplementary Table S2.

### 2.3. Determination of DNA Methylation of the BRCA1 Gene in HEK293H Cells 

HEK293H cells were grown in DMEM with 10% FBS to near confluence in a 100-mm dish. Cells were then exposed to 5 mM bromate or 10 μM chromate for 24 h. Subsequently, cells were washed by 1× phosphate-buffered saline (PBS) twice and supplied with fresh culture medium for an additional 48 h for recovery, allowing DNA damage repair. Cells were then harvested, and genomic DNA was isolated according to the protocol provided by the Promega genomic DNA isolation kit. Genomic DNA was then subject to bisulfite conversion with the Bisulfite Lightning Conversion kit purchased from ZYMO Research. The promoter and transcribed regions of the BRCA1 gene ranging from −189 to +27 were amplified by PCR with the primers shown in Supplementary Table S3. The PCR products were then cloned into the TA vector and sequenced at Florida International University (FIU) DNA Sequencing Core using the BigDye kit purchased from Thermo Fisher Scientific (Waltham, MA, USA). The percentage of DNA methylation and demethylation, as well as the percentage of point mutations at the CpGs were calculated based on the DNA sequencing results from at least 50 colonies.

### 2.4. Micro-Irradiation and Immunofluorescence

One day before transfection, 2 × 10^5^ HEK293H cells were seeded in 2 mL of growth medium on 35-mm glass-bottomed Petri dishes containing an etched grid (MatTek, Ashland, MA, USA). For transfection, 2 μg of pol β-mCherry DNA was diluted in 50 μL of Opti-MEM medium and mixed gently. Lipofectamine™ 2000 (Invitrogen, Grand Island, NY, USA) was diluted by adding 6 μL to 50 μL of Opti-MEM and incubated for 5 min at room temperature. The diluted DNA was combined with diluted Lipofectamine™ 2000, mixed gently, and incubated for 20 min at room temperature. The complex mix was added to a dish of cells in growth medium supplemented with 10 μM BrdU and mixed gently and then incubated at 37 °C in a 5% CO_2_ incubator for 24 h. 

After the addition of fresh room temperature medium, oxidized DNA base lesions, but not double-strand breaks, were specifically introduced by micro-irradiation with a fiber-coupled 355-nm Coherent laser via C-Apochromat 40X/1.20 W Korr FCS M27 objective. The irradiation strip size, 0.208 μm, was manually drawn across the nucleus. Cells were fixed in 4% paraformaldehyde from 1 to 7 min after irradiation, then permeabilized with 0.25% Triton X-100 in PBS for 10 min, washed three times with PBS, and then blocked with 3% bovine serum albumin (BSA) in PBS for 1 h. Cells were then incubated with an anti-8-oxoG antibody (1:50; Santa Cruz Biotech, sc-130914) at 4 °C overnight. Cells were washed three times with PBS and then incubated with m-IgGκ BP-FITC (1:100, Santa Cruz Biotech, sc-516140) for 1 h, and cells were again washed three times with PBS and stained with NucBlue (Invitrogen, Grand Island, NY, USA). Irradiated cells were localized in the dish by grid position. Fluorescence images were acquired with 512 × 512 pixels, with resolution 0.208 µm in bidirectional mode averaging of 4 lines, zoom 2.0, and pixel dwell time 3.15 µs with C-Apochromat 40X/1.20 W Korr FCS M27 objective on the Zeiss LSM780 microscope controlled by Zen 2012 SP5 software.

The recruitment of DNMT3b to 8-oxoGs in cells was detected using immunofluorescence and confocal microscopy. In 200-µl culture medium, 2 × 10^5^ HEK293H cells were seeded in an 8-well chamber slide (1 µ-Slide 8 Well, IbiTreat, ibidi GmbH, Martinsried, Germany). Cells were then subject to the treatment of 5 mM bromate for 2 h. Cells were fixed using 4% paraformaldehyde in PBS at pH 7.4 for 30 min at room temperature and washed with PBS for three times. Cells were then permeabilized for 10 min with PBS containing 0.1% Triton X-100 and washed with PBS for three times. Cells were incubated with blocking buffer containing 1% BSA and 22.52 mg/mL glycine in PBST for 30 min and were incubated with anti-8-oxoG antibody (1:50, Santa Cruz Biotech, sc-130914) and anti-DNMT3b antibody (1:200, Cell Signaling, 67259T) in PBST with 1% BSA overnight at 4 °C. Cells were subsequently washed three times with PBS and incubated with an anti-mouse or anti-rabbit secondary antibody conjugated with Alexa-Fluor^®^-594 (1:1000, Abcam, ab150116) or Alexa-Fluor^®^-488 (1:1000, Abcam, ab150077) in 1% BSA in PBS at room temperature for 1 h. Cells were washed three times with PBS for 5 min in the dark and incubated with 5 µg/mL DAPI in PBS for 1 min. Fluorescence images were acquired using a Nikon C1 confocal microscope equipped with a 60× objective. To determine subcellular localization, Z-stacks were acquired with a 0.4-µm step size set using the Nikon NIS Elements software. 

### 2.5. Detection of the Recruitment of pol β and DNMTs to the BRCA1 Gene by Chromatin Immunoprecipitation (ChIP) 

HEK293H cells were treated with 5 mM bromate or 10 μM chromate for 2 h. Untreated cells were used as the negative control. Following treatment, cells were washed twice with PBS and cross-linked with 1% formaldehyde in complete culture medium (DMEM with 10% FBS) at 37 °C for 30 min. Cross-linking was quenched by the addition of 1 M glycine to a final concentration of 125 mM and incubation with shaking for 5 min at room temperature. Cells were then pelleted by centrifugation at 2000 rpm at 4 °C for 4 min and washed twice in ice-cold PBS containing protease inhibitors (Roche Diagnostics, Indianapolis, IN, USA). Washed cell pellets were resuspended in lysis buffer containing 1% (*w*/*v*) sodium dodecyl sulfate (SDS), 10 mM EDTA, 50 mM Tris-HCl, pH 8.0, and protease inhibitors. Cell suspensions were incubated on ice for 10 min to release cross-linked chromatin before sonication. Cell lysates were further subject to sonication for 12 cycles of 30 s on and 30 s off at 4 °C with Bioruptor ultrasonicator (Diagenode, Denville, NJ, USA). The supernatant of the sheared cell lysate was separated from cell debris by centrifugation at 13,000 rpm for 10 min at 4 °C. The supernatant was then diluted 10-fold with ice-cold ChIP dilution buffer containing 1% (*v*/*v*) Triton X-100, 1.2 mM EDTA, 167 mM NaCl, and 16.7 mM Tris-HCl, pH 8.0 with protease inhibitors. One hundred µl of the diluted supernatant was set aside as input for total DNA control. The remaining lysates were initially incubated with sheared salmon sperm DNA-coated protein A sepharose (Life Technologies, Grand Island, NY, USA) for 2 h at 4 °C with rotation. Subsequently, the lysates were divided into equal aliquots as the IgG control and immunoprecipitates (IP) with pol β, DNMT3b, and DNMT1, respectively. For each IP, the diluted chromatin solutions were incubated overnight with 3 μg rabbit anti-pol β antibody (ab26343, Abcam, Cambridge, MA, USA), 3 μg rabbit anti-DNMT3b antibodies (ab2851, Abcam, Cambridge, MA, USA), or 3 μg rabbit anti-DNMT1 antibodies (ab13537, Abcam, Cambridge, MA, USA) at 4 °C with rotation. Subsequently, IPs for pol β, DNMT3b, and DNMT1 and the IgG control were incubated with sheared salmon sperm DNA coated protein A sepharose beads for 2 h at 4 °C with rotation. IPs that bound to protein A sepharose beads were pelleted with centrifugation at 1000 rpm for 2 min and washed two times with low-salt washing buffer that contained 150 mM NaCl, 0.1% (*w*/*v*) SDS, 1% (*v*/*v*) Triton X-100, 2 mM EDTA, and 20 mM Tris-HCl, pH 8.0, followed by high-salt washing buffer that contained 500 mM NaCl, 0.1% (*w*/*v*) SDS, 1% (*v*/*v*) Triton X-100, 2 mM EDTA, and 20 mM Tris-HCl, pH 8.0 and washed by TE buffer. IPs were then eluted from agarose beads by incubation with a freshly prepared elution buffer that contained 1% (*w*/*v*) SDS and 0.1 M NaHCO_3_. Subsequently, IPs were subject to cross-linking reversal with 0.2 M NaCl and incubated at 65 °C overnight. Released DNA was cleaned with proteinase K digestion at 45 °C for 2 h and phenol/chloroform extraction. DNA was then recovered by ethanol precipitation. The precipitated DNA was dissolved in TE buffer and used for subsequent quantitative PCR. Quantitative PCR was performed using SYBR Green Supermix (Bio-Rad Laboratories Hercules, CA, USA) in a 20 µL reaction mixture according to the manufacturer’s protocols. Samples were amplified using a CFX Connect Real-Time PCR Detection System from Bio-Rad Laboratories (Hercules, CA, USA). The sequences of the PCR primers are listed in Supplementary Table S3. Ct values that were recorded in CFX Manager Software (Bio-Rad Laboratories, Hercules, CA, USA) during PCR were used for quantifying data to evaluate the fold difference between experimental samples and the normalized input as described previously [35]. The “% Input” value represents the enrichment of pol β, DNMT3b, and DNMT1 on the BRCA1 gene. Statistical analysis was performed with GraphPad Prism 6 (GraphPad Software, San Diego, CA, USA). Statistically significant differences in the data were tested by standard two-way analysis of variance with Tukey’s multiple comparison posttests. A significant difference was designated at *P* < 0.05.

### 2.6. Detection of the Interaction between pol β and DNMTs by Co-Immunoprecipitation (co-IP) and Immunoblotting

The interaction between pol β and DNMTs, i.e., DNMT3b and DNMT1, was examined by employing co-IP. HEK293H cells were treated with 5 mM bromate or 10 μM chromate for 2 h. Cells were harvested in cold PBS buffer. Cell lysates were prepared as described previously [35] and incubated with 50 µl Protein A/G PLUS-agarose (Santa Cruz Biotechnology, Inc., Dallas, Texas, USA) at 4 °C for 2 h with rotation. Cell lysates were further incubated without or with 3 μg of rabbit anti-pol β antibodies (ab26343, Abcam, Cambridge, MA, USA), 3 μg of rabbit anti-DNMT3b antibody (ab2851, Abcam, Cambridge, MA, USA), 3 μg of mouse anti-DNMT1 antibodies (ab13537, Abcam, Cambridge, MA), or 3 μg of rabbit IgG (ab37451, Abcam, Cambridge, MA, USA) at 4 °C overnight with rotation, respectively. Cell lysates were then incubated with 60 μL Protein A/G PLUS-agarose at 4 °C for an additional 2 h with rotation followed by three washes at 4 °C in washing buffer containing 20 mM HEPES, pH 7.5, 50 mM NaCl, 1% NP-40, and 2 mM EDTA. DNase I digestion of DNA in cell lysates was performed before immunoprecipitation using 5 units of RNase-free DNase I (Thermo Fisher Scientific, Waltham, MA, USA) in the presence of 2.5 mM MgCl_2_ at 37 °C for 30 min and then terminated by 10 mM EDTA. The antibody-protein complexes were eluted by heating in SDS loading buffer at 50 °C for 10 min and subsequently subject to SDS-PAGE and immunoblotting with rabbit anti-human pol β antibody (1:1,000; ab175197, Abcam, Cambridge, MA, USA), mouse anti-DNMT3b antibody (1:100; sc-376043, Santa Cruz Biotechnology, Dallas, Texas, USA), or rabbit anti-DNMT1 antibody (1:1000; ab19905, Abcam, Cambridge, MA, USA) followed by incubation with goat anti-rabbit IgG (1:10,000; ab6721, Abcam, Cambridge, MA, USA) or rabbit anti-mouse IgG (1:10,000; ab6728, Abcam, Cambridge, MA, USA) along with chemiluminescent substrates (Pierce-Thermo Scientific, Rockford, lL, USA). 

### 2.7. Measurement of pol β DNA Synthesis in Bypassing an 8-OxoG and Nucleotide Misinsertion 

Pol β DNA synthesis in bypassing an 8-oxoG adjacent to a normal C or a 5mC in the BRCA1 promoter was performed by incubating purified human pol β with the substrates bearing an 8-oxoG opposite the C without or with a 5mC located at −166 nt of the promoter strand of the BRCA1 gene. Pol β (50 nM) was incubated with 25 nM substrates in 10 μL reaction mixture containing BER reaction buffer, 5 mM MgCl_2_, and 50 μM dNTPs at 37 °C for 30 min. Pol β nucleotide misinsertion was measured by incubating pol β (50 nM) with 25 nM substrates containing an 8-oxoG without or with a 5mC on the template strand. The 10-μL reaction mixture was assembled in BER reaction buffer containing 5 mM MgCl_2_ and 50 μM A, T, G, or C. BER reactions were assembled on ice and incubated at 37 °C for 30 min. Reactions were terminated with 2× stopping buffer containing 95% formamide and 10 mM EDTA followed by incubation at 95 °C for 5 min. All the substrates were ^32^P-labeled at the 5′-end of the upstream strand. Substrates and products were separated with 15% or 18% urea-denaturing polyacrylamide gel electrophoresis and detected with the Pharos FX Plus PhosphorImager (Bio-Rad, Hercules, CA, USA).

## 3. Results

### 3.1. Oxidative DNA Damage Altered the DNA Methylation Pattern of the BRCA1 Gene

**Figure 1:**
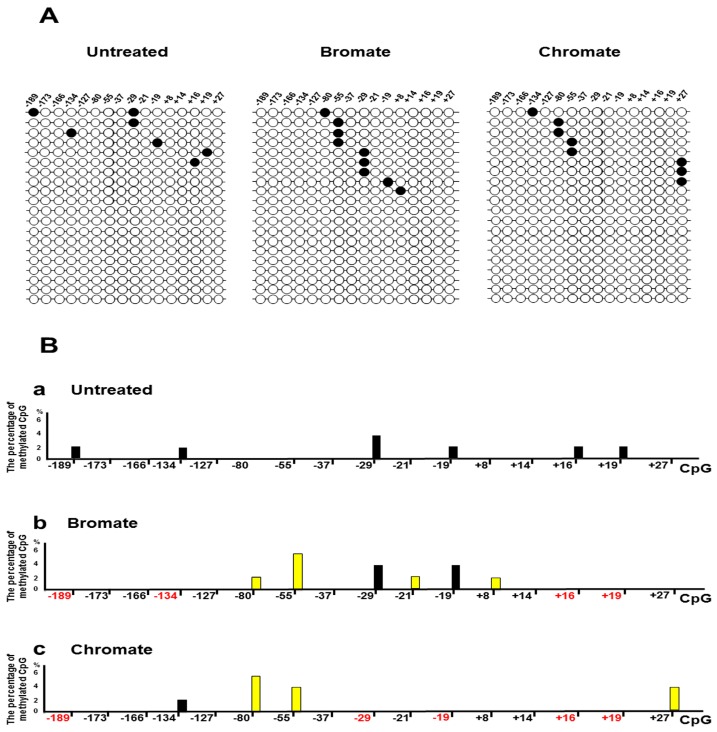
Oxidative DNA damage modulates the DNA methylation pattern of the promoter and transcribed regions of the breast cancer 1 (BRCA1) gene in cells: HEK293H cells were treated with bromate or chromate, as described in the Materials and Methods section. Subsequently, genomic DNA was isolated from the cells and subject to bisulfite sequencing. (**A**) Representative results of the DNA methylation pattern of the BRCA1 promoter and transcribed regions (−189 to +27) are illustrated. The panel on the left indicates the results of the untreated cells. The panel in the middle represents the results from the cells treated with 5 mM bromate for 24 h with recovery for 48 h. The panel on the right illustrates the results from the cells treated with 10 μM chromate for 24 h with recovery for 48 h. (**B**) The percentage of methylated CpGs: Panels a, b, and c illustrate the percentage of methylated CpGs in the promoter and transcribed regions of BRCA1 gene in the untreated cells, cells treated with 5 mM bromate for 24 h with 48 h recovery, and cells treated with 10 μM chromate for 24 h with 48 h recovery, respectively. The bars in yellow indicate the new DNA methylation sites induced by bromate or chromate. The percentage of methylated CpGs was calculated from the DNA sequencing results from at least 50 colonies.

**Figure 2:**
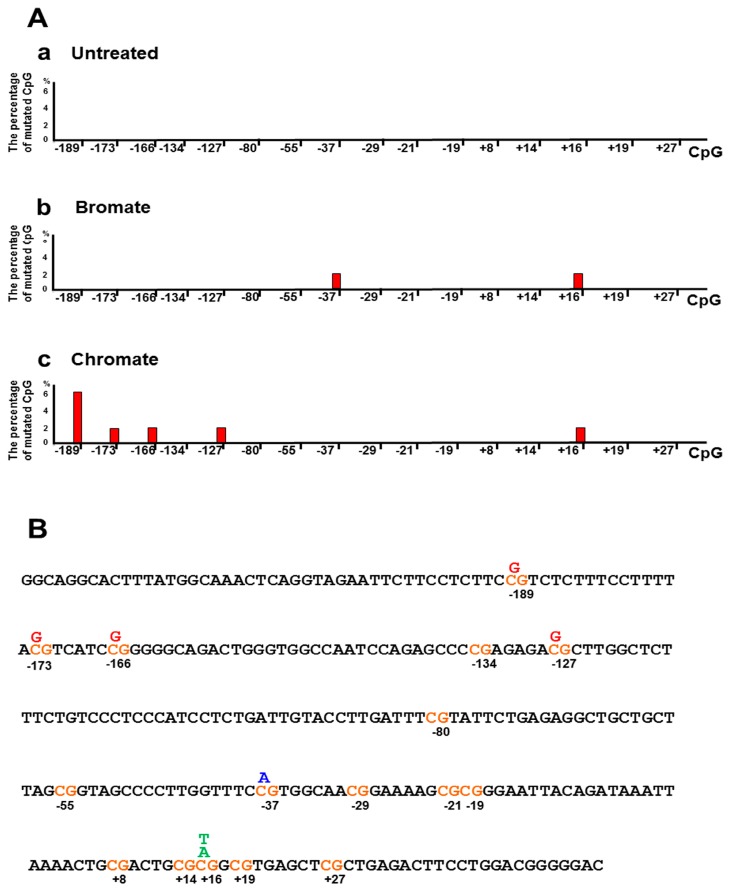
Oxidative DNA damage induces mutations in the promoter and transcribed regions of the BRCA1 gene in cells. (**A**) The percentage of the mutations (bars in red) in the promoter and transcribed regions of the BRCA1 gene induced by oxidative DNA damage from the treatment of bromate (5 mM) and chromate (10 µM). (**B**) The mutation spectrum in the BRCA1 gene is illustrated. The mispaired nucleotides in blue were induced by bromate treatment, whereas misincorporated nucleotides in red were induced by chromate treatment. The mispaired nucleotides in green were generated from the treatment of both the bromate and chromate. The percentage of point mutations at the CpGs was calculated from the DNA sequencing results from at least 50 colonies.

Since oxidative DNA damage can oxidize cytosine, 5mC, and guanine, it is possible that damaged DNA bases and the resulted single-strand DNA (ssDNA) breaks at the CpGs of the BRCA1 gene may alter the DNA methylation pattern by inducing the loss of 5mCs and point mutations. To test this possibility, we initially determined the DNA methylation pattern on the promoter and transcribed regions of the BRCA1 gene in the untreated HEK293H cells and cells treated with oxidative DNA damaging agents bromate (5 mM) and chromate (10 μM) using bisulfite DNA sequencing (Figure 1). These agents result in 8-oxoG and ssDNA breaks [36,37]. The results showed that bromate and chromate treatment led to a unique DNA methylation pattern in the promoter and transcribed regions of the BRCA1 gene, which is significantly different from that in the untreated cells (Figure 1A). In the untreated cells, the CpGs located at −189, −134, −29, −19, +16, and +19 of the gene are methylated (2–3%) (Figure 1B, panel a). However, bromate treatment (5 mM) for 24 h with 48 h recovery resulted in the loss of methylation at −189, −134, +16, and +19 (Figure 1A, the panel in the middle; Figure 1B, panel b, the CpG sites in red). Surprisingly, bromate also led to the formation of new methylation sites at the CpGs located at −80, −55, −21, and +8 (2–4%) (Figure 1A,B, bars in yellow). Chromate at 10 µM for 24 h with 48 h recovery led to the loss of DNA methylation at −189, −29, −19, +16, and +19 (Figure 1B, panel b, the CpG sites in red) and to the formation of new methylation sites at −80, −55, and +27 (2–4%) (Figure 1A, the panel on the right; Figure 1B, panel c, bars in yellow). Further mutational analysis showed that bromate induced C to A and C to T mutations at CpG located at −37 and +16 (2%) (Figure 2) and that chromate mainly led to C to G mutations at −189, −173, −166, −127, and +16 (2–6%) (Figure 2). Interestingly, we found that, although CpGs at −80, −55, and +27 were methylated upon oxidative DNA damage, no mutations at the sites were detected. This may suggest that DNA methylation at these CpGs recruited methylation-binding proteins and other proteins preventing the DNA damage agents from accessing the CpGs and mutations.

### 3.2. Pol β Is Recruited to Oxidative DNA Damage and Simultaneously Recruited to the Promoter and Transcribed Regions of the BRCA1 Gene with DNMT3b upon Oxidative DNA Damage in HEK293H Cells

**Figure 3:**
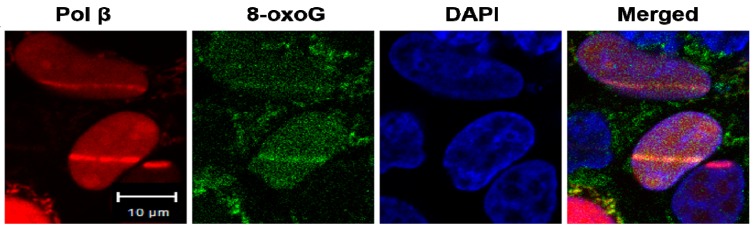
Pol β is recruited to 8-oxoGs in cells: Micro-irradiation was employed to induce 8-oxoGs in HEK293H cells that were transfected with a plasmid expressing mCherry-tagged human pol β. Pol β-mCherry was recruited (red), and 8-oxoG stained with an anti-8-oxoG antibody (green) was generated 4 min after micro-irradiation damage. The nucleus was stained with DAPI (blue). The experiments were done in triplicate.

To further determine whether bromate- and chromate-induced oxidative DNA damage resulted in the loss of 5mCs, mutations, and new methylation sites at the BRCA1 gene through pol β during BER, we initially analyzed the recruitment of pol β to the oxidized DNA bases, 8-oxoGs that can be induced by bromate and chromate. HEK293H cells transfected with a plasmid expressing mCherry-tagged human pol β were micro-irradiated to create 8-oxoGs. Pol β-mCherry was recruited to the same nuclear DNA damage site stained for 8-oxoGs with an anti-8-oxoG antibody (green) (Figure 3). This suggests that pol β-mCherry was recruited to 8-oxoGs in cells (Figure 3).

**Figure 4:**
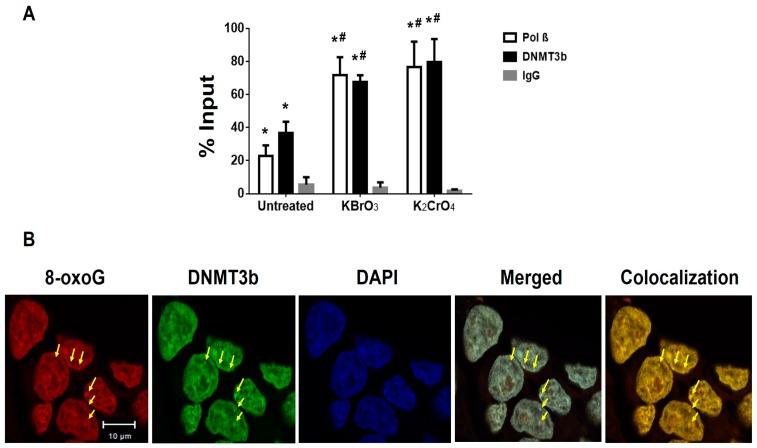
The recruitment of pol β and DNA methyltransferase 3b (DNMT3b) to the promoter and transcribed regions of the BRCA1 gene upon oxidative DNA damage: (**A**) The recruitment of pol β and DNMT3b to the promoter and transcribed regions of the BRCA1 gene was detected by Chromatin Immunoprecipitation (ChIP) assay as described in the Materials and Methods section. The quantification of the amount of DNA that represents the amount of pol β and DNMT3b recruited to the regions of the BRCA1 gene in the untreated cells and cells treated with 5 mM bromate or 10 µM chromate was shown. The “% Input” was calculated using the following equation: Input % = 2^−ΔCt (normalized ChIP)^ × 100. The results were obtained from three independent experiments and illustrated as mean ± S.D. Two-way ANOVA with Tukey’s multiple comparison posttests was used to determine statistically significant differences. “*” denotes *P* < 0.05 compared to the IgG control, and “#” denotes *P* < 0.05 compared with the untreated cells. (**B**) The recruitment of DNMT3b to 8-oxoGs in cells was detected using immunofluorescence and confocal microscopy. 8-oxoGs (red) induced by 5 mM bromate for 2 h was stained by the same anti-8-oxoG antibody as the one shown in Figure 3. DNMT3b (green) was stained with an anti-DNMT3b antibody. The nucleus was stained with DAPI (blue). The arrows indicate the foci of the recruitment of DNMT3b to 8-oxoG sites. The experiments were done in triplicate.

Since previous studies have shown that oxidative DNA damage can induce the recruitment of DNMTs that include both DNMT1 and DNMT3b to DNA damage sites [38], we then examined the recruitment of pol β, DNMT1, and DNMT3b to the promoter and transcribed regions of the BRCA1 gene using ChIP assay (Figure 4 and Supplementary Figure S1). The proteins were recruited to the BRCA1 gene in the untreated cells (Figure 4 and Supplementary Figure S1). However, only the recruitment of pol β and DNMT3b to the BRCA1 gene was significantly increased upon the treatment of bromate and chromate (Figure 4A), indicating that the proteins were concomitantly recruited to the BRCA1 gene in a damage-dependent manner. This further suggests that pol β may interact with DNMT3b to facilitate the recruitment of DNMT3b to the damaged sites. This is further supported by the results showing that DNMT3b was also recruited to 8-oxoG sites induced by bromate (Figure 4B). Similarly, the recruitment of pol β and DNMT3b to the β-actin gene was also significantly increased upon the treatment of bromate and chromate (Supplementary Figure S2), indicating that the proteins were also recruited to the β-actin gene upon oxidative DNA damage. This further suggests that pol β and DNMT3b can be recruited to the other regions in the genome upon oxidative DNA damage to impact DNA methylation patterns globally. In contrast, the recruitment of DNMT1 to the BRCA1 gene was independent of DNA damage induced by bromate and chromate (Supplementary Figure S1), suggesting that DNMT1 was not involved in the DNA methylation induced by the oxidative DNA damage induced by bromate and chromate.

### 3.3. Pol β Interacts with DNMT3b in HEK293H Cells

**Figure 5:**
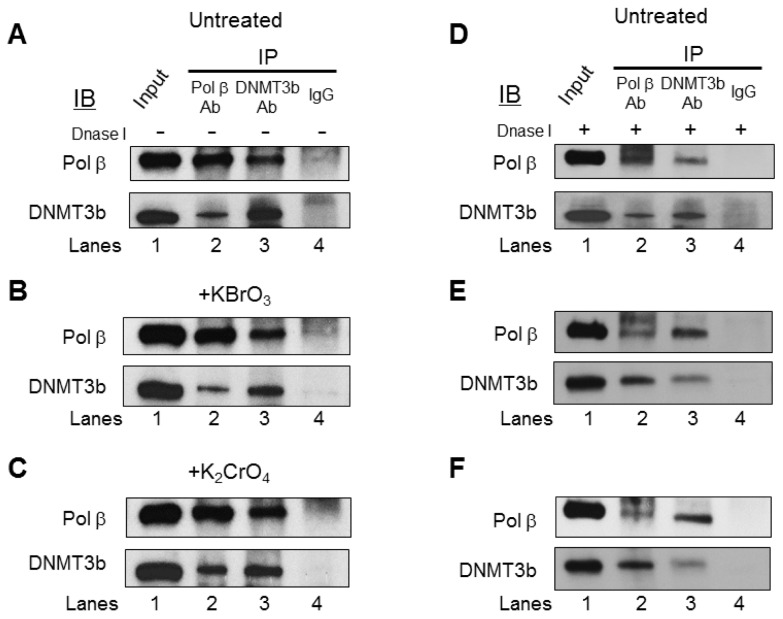
Pol β interacts with DNMT3b in cells. Co-IP and immunoblotting (IB) of pol β or DNMT3b in cell lysates was performed without (**A**–**C**) and with (**D**–**F**) DNase I digestion as described in the Materials and Methods section: The lysates were prepared from the untreated HEK293H cells (**A**,**D**) or cells treated with 5 mM bromate (**B**,**E**) or 10 μM chromate (**C**,**F**) for 2 h. Cell lysates were subject to co-IP and IB for pol β and DNMT3b, as described in the Materials and Methods section. Lane 1 corresponds to the whole lysates of the untreated cells designated as the “Input” control. Lanes 2 and 3 correspond to cell lysates immunoprecipitated with an anti-pol β and an anti-DNMT3b antibody, respectively. Lane 4 is the cell lysates immunoprecipitated with rabbit IgG alone. IP represents an immunoprecipitation; IB indicates immunoblotting. Experiments were done in triplicate.

Since pol β and DNMT3b were concomitantly recruited to the BRCA1 gene (Figure 4), we then reasoned that pol β and DNMT3b might interact with each other to coordinate DNA base lesion repair and modulation of DNA methylation at the promoter and transcribed regions of the BRCA1 gene in cells. To test this possibility, we examined if pol β and DNMT3b can interact to form a protein complex in cells using co-IP. We found that, in both the untreated (Figure 5A) and treated cells (Figure 5B,C), DNMT3b proteins were detected in the anti-pol β immunoprecipitates (IPs) (Figure 5A–C, lane 2) and that pol β proteins were also detected in the anti-DNMT3b IPs (Figure 5A–C, lane 3). IgG failed to precipitate pol β or DNMT3b from the cell extracts (Figure 5, IP IgG, lane 4). Both proteins were detected in the cell extract input (Figure 5, lane 1). Moreover, the interaction between pol β and DNMT3b was detected in the cell lysates that were subject to DNase I digestion (Figure 5E,F), demonstrating that the protein-protein interaction was not mediated by the presence of DNA. The results indicate that pol β and DNMT3b constitutively interacted with each other in cells (Figure 5). Although we detected a small amount of DNMT1 proteins in the anti-pol β IPs (Supplementary Figure S3, lane 2), suggesting a weak interaction between pol β and DNMT1 in cells, the ChIP results showed that the recruitment of DNMT1 to the regions of the BRCA1 gene was independent of oxidative DNA damage (Supplementary Figure S1). This indicates that pol β and DNMT1 were not simultaneously recruited to the DNA damage sites induced by bromate and chromate in the BRCA1 gene. This further indicates that the pol β cooperated with DNMT3b rather than DNMT1 during BER of oxidative DNA damage in cells.

### 3.4. Pol β Bypasses an 8-oxoG in the BRCA1 Gene through Nucleotide Misinsertion

**Figure 6:**
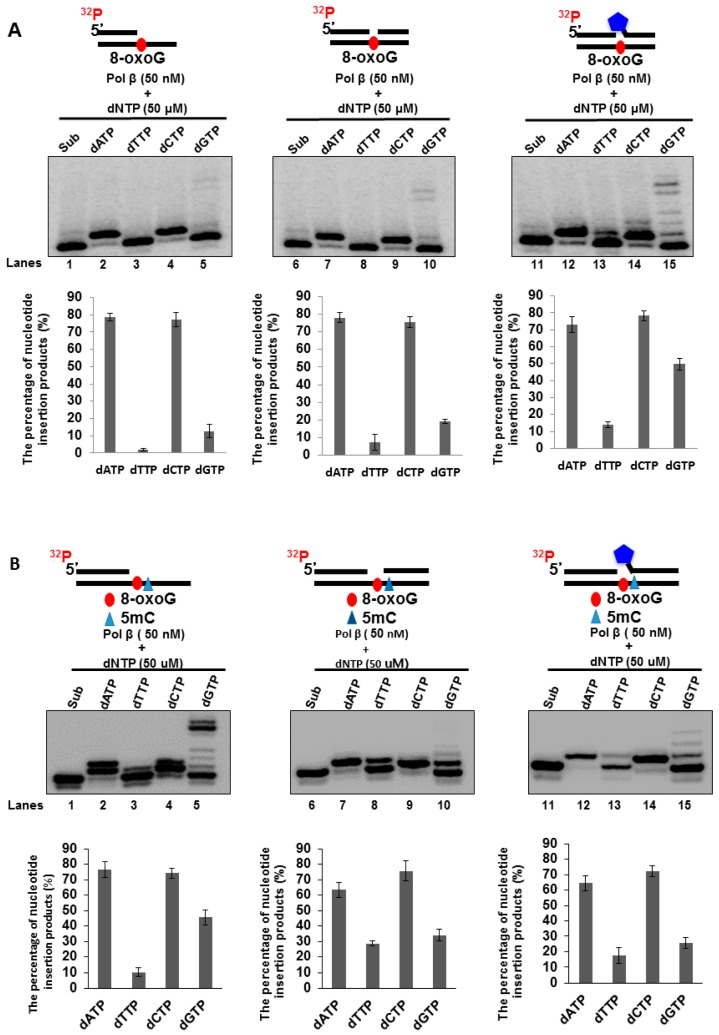
Pol β nucleotide insertion for bypassing an 8-oxoG: Bypass of an 8-oxoG by pol β was examined by incubating the open-template substrate and 1-nt gap substrates that contain a downstream 5′-phosphate or 5′-tetrahydrofuran (THF) residue and a template strand with 8-oxoG (**A**) and with both 5mC and 8-oxoG (**B**), with 50 nM pol β in the presence of 50 µM dATP or dTTP or dCTP or dGTP, respectively. Lanes 1, 6, and 11 represent substrate only. Lanes 2–5, 7–10, and 12–15 correspond to reaction mixtures with 50 nM pol β in the presence of 50 µM dATP or dTTP, or dCTP or dGTP. Substrates were ^32^P-labeled at the 5′-end of the upstream primer as indicated. Substrates are illustrated schematically above the gel. All experiments were done in triplicate. Quantification of the results is shown below the gels.

Previous studies have shown that, during DNA replication and BER, pol β can bypass a DNA base lesion [39,40,41,42,43,44,45,46,47]. Furthermore, pol β lesion bypass can induce a high frequency of mutations in the genome [39,40,41,42,43,47,48,49,50,51]. Also, since pol β does not have a 3′–5′ exonuclease activity for proofreading, a high level of pol β can result in nucleotide misincorporation, leading to gene mutations and cancer development [52,53,54]. Thus, we hypothesized that a high level of pol β might be recruited to 8-oxoGs in the BRCA1 gene to bypass the lesions through its translesion synthesis activity leading to the point mutations at the CpG sites of the gene. To test this, we examined if pol β can bypass an 8-oxoG through nucleotide misinsertion to create a G:G mispair at the promoter region of the BRCA1 gene. This was determined by incubating 50 nM purified pol β protein with an open-template or gapped substrate containing an 8-oxoG at the template strand of the CpG located at −166 of the human BRCA1 gene promoter. We found that 50 nM pol β inserted dA, dC, and dG to base pair with the 8-oxoG on the open-template substrate and 1-nt gap substrate (Figure 6A, lanes 2, 4, 5, 7, 9, and 10) but with a higher efficiency in inserting dA and dC (Figure 6A, lanes 2, 4, 7, and 9). For the 1-nt gap-THF substrate containing an 8-oxoG, 50 nM pol β mainly inserted dA and dC to bypass the lesion (Figure 6A, lanes 12 and 14). It also inserted dT and dG with relatively low efficiency (Figure 6A, lanes 13 and 15). Interestingly, we found that pol β inserted dG to bypass the 8-oxoG in all the substrates (Figure 6A, lanes 5, 10, and 15). In addition, pol β exhibited a higher efficiency to insert a dG with the 1-nt gap-THF substrate than the open-template and 1-nt gap substrate (Figure 6A, compare lane 15 with lane 5 and lane 10). The results indicate that pol β misinserted dG and dA to bypass an 8-oxoG during BER in CpGs. This is consistent with our results showing that oxidative DNA damage predominantly induced C to G and C to A mutations via G:G and A:G mismatches (Figure 2B) through pol β nucleotide misincorporation.

We further tested the effect of 5mC on the pol β bypass of 8-oxoG. The results showed that Pol β exhibited a similar pattern of nucleotide insertion to bypass 8-oxoG adjacent to 5mC compared with that of 8-oxoG alone for all the substrates (compare Figure 6A,B). However, in the open template substrate containing 5mC and 8-oxoG, Pol β insertion of G to bypass the damaged base was increased by ~4-fold (compare lane 5 of Figure 6B with that of Figure 6A). Interestingly, Pol β insertion of G to bypass 8-oxoG on the 1nt-gap substrate containing a THF with both 5mC and 8-oxoG was decreased by ~2.5-fold (compare lane 15 of Figure 6B with that Figure 6A). The results suggest that 5mC at the 5’ side of the 8-oxoG facilitated C to G mutation by Pol β on the open template DNA but inhibited C to G mutation on the 1nt gap with a THF residue.

## 4. Discussion

**Figure 7:**
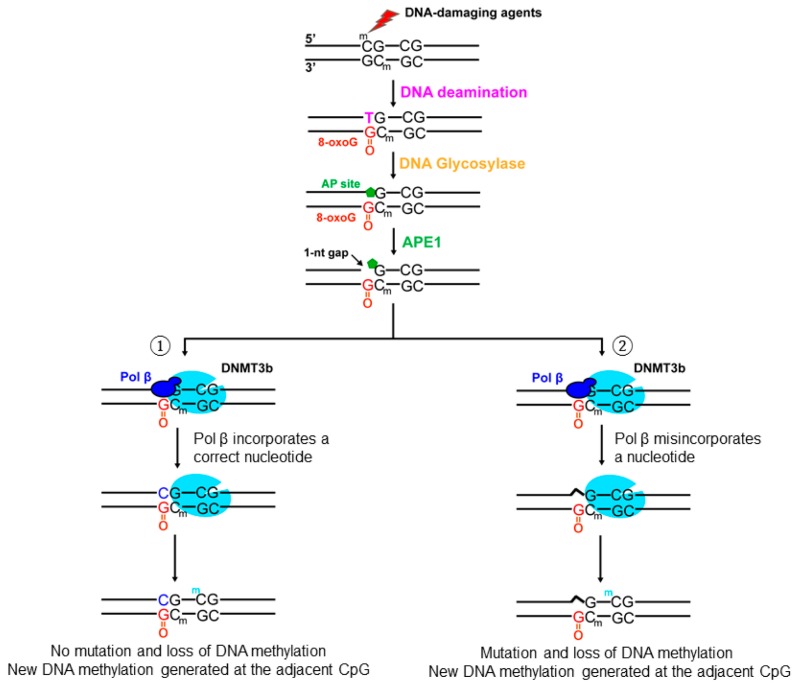
Oxidative DNA damage alters DNA methylation patterns in the promoter and transcribed regions of the BRCA1 gene through the crosstalk between pol β and DNMT3b. Oxidized DNA base lesions such as 8-oxoGs can occur at the CpGs located in the promoter and transcribed regions of the BRCA1 gene. The lesions can be removed by a DNA glycosylase, leaving an abasic (AP) site. The AP site is subsequently cleaved by APE1, generating the 1-nt gap intermediates. Subsequently, pol β is recruited and performs nucleotide insertion. In the scenario where pol β inserted a correct nucleotide to bypass an 8-oxoG, DNMT3b is recruited to the CpG dinucleotides through its interaction with pol β. This allows DNMT3b to create a new 5mC on the adjacent CpG site but not at the damaged site because of the inhibition of the methyltransferase activity by the base lesion. This then results in the loss of 5mC and a new methylation site (subpathway 1). In the scenario where pol β inserted an incorrect nucleotide to bypass an 8-oxoG, DNMT3b is also recruited to the damage site through its interaction with pol β leading to a mutation, loss of 5mC, and addition of a new 5mC at the adjacent CpG site (subpathway 2).

In this study, for the first time, we demonstrated that oxidative DNA damage altered the DNA methylation pattern of the BRCA1 gene by inducing the loss of 5mCs, point mutations, and formation of new 5mCs (Figure 1 and Figure 2). We showed that oxidative DNA damage induced the loss of 5mCs on the CpGs located at −134, −29, −19, +16, and +19 and point mutations on the CpGs at −189, −173, −166, −127, −37, and +16 (Figure 1 and Figure 2). We found that oxidative DNA damage predominantly caused C to G and C to A mutations along with C to T mutation (Figure 2) and that this was mediated by pol β nucleotide misinsertion in bypassing an 8-oxoG (Figure 6). We demonstrated that oxidative DNA damage created new methylation sites on the CpGs located at −80, −55, −21, +8, and +27 (Figure 1B, bars in yellow) that are adjacent to the sites with the loss of 5mCs and point mutations (Figure 1 and Figure 2). We showed that a high level of pol β was recruited to 8-oxoGs induced by micro-irradiation in the cells (Figure 3) and that pol β interacted with DNMT3b but not DNMT1 in the BRCA1 gene promoter and transcribed regions (Figure 4 and Figure 5 and Supplementary Figure S1). The results support a hypothetical model where pol β is recruited to oxidative DNA damage on the CpGs at the BRCA1 gene to perform its gap-filling synthesis. This subsequently leads to the loss of 5mCs via substitution of a 5mC with C or point mutations at the CpGs through pol β nucleotide misinsertion while it bypasses an 8-oxoG on the template strand (Figure 7, subpathways 1 and 2). Simultaneously, pol β interacts with DNMT3b, and subsequently, DNA methyltransferase binds to the CpGs adjacent to damaged bases, leading to the generation of new methylated CpGs on the BRCA1 gene (Figure 7, subpathways 1 and 2). 

Here, we provided the first evidence showing that a repair DNA polymerase, pol β, was simultaneously recruited with the *de novo* DNA methyltransferase, DNMT3b, to the BRCA1 gene through its interaction with the DNA methyltransferase to repair a base lesion and to create new 5mCs on the CpGs sites adjacent to the base lesion. We showed that pol β was recruited to 8-oxoGs in cells and subsequently interacted with DNMT3b to recruit the DNA methyltransferase to the region adjacent to the oxidized DNA bases leading to *de novo* DNA methylation at the adjacent CpGs. We further demonstrated that oxidative DNA damage induced the loss of DNA methylation sites through nucleotide misincorporation by pol β. Thus, our results provide new mechanistic insights into how oxidative DNA damage can induce new DNA methylation sites to modulate the DNA methylation pattern of the BRCA1 gene through the crosstalk between pol β and a *de novo* methyltransferase during BER. Our results further support the conclusion that oxidative DNA damage resulted in the dynamic changes of DNA methylation in the BRCA1 gene through pol β nucleotide misincorporation and the crosstalk between pol β and DNMT3b. Considering the fact that oxidative DNA damage can be constitutively generated on CpGs that spread in a genome-wide fashion and that pol β and DNMT3b interacted with each other upon the DNA damage in cells (Figure 5), our results suggest that pol β and DNMT3b can also be simultaneously recruited to all the regions in the genome that contain oxidative DNA damage to alter DNA methylation patterns in a DNA sequence-specific manner. This is further supported by the results showing that the recruitment of pol β and DNMT3b to the β-actin gene was significantly increased upon oxidative DNA damage (Supplementary Figure S2). The roles of the crosstalk between pol β and DNMT3b in modulating the methylation of CpGs in other genes and sequences in the genome needs to be further studied. It is also of importance to further elucidate if other DNA repair polymerases may also coordinate with DNMT3b to modulate DNA methylation during BER of oxidative DNA damage in the future.

It has been reported that the mismatch repair proteins, MSH2 and MSH2-MSH6, can be recruited to oxidative DNA damage to recruit DNMT1 to the damaged sites, causing DNA methylation pattern changes [55,56]. This suggests that mismatch repair proteins can mediate the recruitment of DNMTs to DNA base lesions. Interestingly, a recent study from our group has demonstrated that MSH2-MSH3 can physically interact with pol β in cells [35]. Thus, it is possible that mismatch proteins may also recruit DNMT3b to the oxidative DNA damage sites through their interactions with pol β to induce the aberrant *de novo* DNA methylation patterns in the BRCA1 gene associated with deregulation of the gene and tumorigenesis. An interplay among pol β, mismatch repair proteins, and DNMTs during BER needs to be further explored. 

Recent reports have also shown that oxidative DNA damage induced by hydrogen peroxide recruits both DNMT3b and DNMT1 along with histone modification enzymes that include SIRT1 and EZH2 to the GC-rich regions of the genome that include CpG islands at gene promoter regions [38,55]. This suggests that both DNMT3b and DNMT1 might be involved in the methylation pattern change at CpG islands induced by oxidative DNA damage. However, our study demonstrated that DNMT3b rather than DNMT1 was responsible for the formation of new methylation sites that are adjacent to oxidized DNA bases at the BRCA1 gene. This further indicates that the *de novo* methyltransferase, DNMT3b, can readily modulate DNA methylation pattern through its interaction with pol β during BER of base lesions and ssDNA breaks compared with the maintenance methyltransferase, DNMT1, which requires hemimethylated CpGs as its substrates [57]. This supports the notion that DNMT1 is involved in double-strand DNA breaks-induced gene silencing [58]. Our results further suggest that pol β serves as a “sensor protein” to recruit DNMT3b to the CpGs adjacent to DNA base lesions and ssDNA breaks to generate new 5mCs at the BRCA1 gene during BER leading to aberrant methylation pattern. 

It is reported that hypermethylation in repeated DNA sequences may be mediated by *de novo* DNA methylation [59,60]. However, the underlying mechanisms remain unknown. Since the tandem repeats are hotspots of oxidative DNA damage, our study suggests that oxidative DNA damage in the repeated sequences can induce *de novo* methylation through the pol β-DNMT3b interaction during BER, thereby leading to hypermethylation in these sequences. The roles of the crosstalk between BER and other DNA repair pathways and *de novo* DNA methylation in the hypermethylation of DNA repeated sequences and other cellular functions need to be further elucidated. It should be noted that the expression and distribution of DNMT1 and DNMT3b are cell -cycle dependent [61,62]. This may affect the interaction of pol β with the DNA methyltransferases. The cell cycle dependency of the interactions between pol β and DNA methyltransferases warrant to be studied in the future.

Our study has revealed a novel mechanism underlying the modulation of DNA methylation patterns induced by oxidative DNA damage at the tumor suppressor BRCA1 gene through the coordination between BER and *de novo* DNA methylation. Previous studies have examined the pol β bypass of an 8-oxoG in a non-gene-related DNA sequence [63,64]. Here, we have further elucidated the pol β bypass of an 8-oxoG at the CpG dinucleotides in the unique DNA sequences of the tumor suppressor BRCA1 gene (Figure 6). Furthermore, it has been shown that 5mC does not affect DNA synthesis by DNA polymerases including pol β [65,66]. In addition, a study from the Wilson group has demonstrated that 5mC in the CpG dinucleotides fails to affect the BER of 8-oxoG that is mediated by pol β [67], indicating that 5mC does not affect pol β bypass of 8-oxoG. However, we found that 5mC stimulated Pol β G:G misincorporation on the open template DNA (Figure 6B, lane 5) but inhibited the nucleotide misincorporation by Pol β on the 1nt gap with a sugar phosphate formed on the RRCA1 gene (Figure 6B, lane 15). It is possible that 5mC adjacent to 8-oxoG stabilizes G to G mismatch by Pol β on the open template DNA whereas 5mC on the 1nt-gap with a sugar phosphate may disrupt G to G mismatch by Pol β misincorporation. 

In this study, HEK293H cells, which represent normal cells, were used for determining the effects of oxidative DNA damage on the DNA methylation pattern of the BRCA1 gene and the underlying mechanisms. Our results provide new mechanistic insights into how oxidative DNA damage can modulate the DNA methylation pattern of the BRCA1 gene through the crosstalk between BER and *de novo* DNA methylation in normal cells. The study has laid a foundation for a future study on how oxidative DNA damage may potentially lead to cancer development by transforming normal cells into cancer cells through alteration of the DNA methylation patterns of a tumor suppressor gene.

Using bisulfite DNA sequencing, we detected aberrant DNA methylation patterns at the BRCA1 gene, i.e., epimutations including hyper- and hypomethylation and point mutations at the CpGs sites. Our results are consistent with an early study indicating that, in normal human mammary epithelial cells and peripheral blood lymphocytes, the CpG sites at the promoter and encoding regions of the BRCA1 gene are unmethlyated with a low percentage of DNA methylation [68]. A recent study also demonstrates a similar percentage of epimutations at the promoter region of the BRCA1 gene as our study in normal human peripheral white blood cells [69] using deep bisulfite sequencing. All these results suggest that different types of cells share the similarity in the DNA methylation pattern and epimutations of the BRCA1 gene induced by oxidative DNA damage.

Our current studies further suggest the important roles of DNA polymerases, including pol β and other DNA polymerases in modulating DNA methylation patterns on a tumor suppressor gene during BER of oxidative DNA damage. Since multiple DNA polymerases can cooperate during BER, it is of interest to elucidate their roles in modulating DNA methylation in tumor suppressor genes with the gene knockdown of the polymerases in human cells. 

## Supplementary Materials

The following are available online at https://www.mdpi.com/2073-4409/9/1/225/s1, Table S1: List of antibodies, Table S2: Oligonucleotides sequences, Table S3: Primer sequences, Figure S1: The recruitment of pol β and DNMT1 to the promoter and transcribed regions of the BRCA1 gene, Figure S2: The recruitment of pol β and DNMT3b to the β-actin gene, Figure S3: Pol β interacts weakly with DNMT1 in cells.
