# Supplementary material for: Oxidative DNA Damage Modulates DNA Methylation Pattern in Human Breast Cancer 1 (BRCA1) Gene via the Crosstalk between DNA Polymerase β and a de novo DNA Methyltransferase
Source: Cells. 2020 Jan 16;9(1):225. doi: 10.3390/cells9010225 (PMC7016758; doi:10.3390/cells9010225)
Supplement: Supplementary file 1 [file cells-09-00225-s001.pdf]

Table S1. List of antibodies

	Antibodies	Application	Supplier	Catalog number	Dilution/ Amount	Species	Type
**Primary Antibodies**	8-oxoG	Immunofluorescence	Santa Cruz Biotechnology	sc-130914	1:50	Mouse	Monoclonal
	DNMT3b	Immunofluorescence	Cell Signaling	67259	1:200	Rabbit	Monoclonal
	Pol β	ChIP, Co-IP	Abcam	ab26343	3 μg	Rabbit	Polyclonal
	DNMT3b	ChIP, Co-IP	Abcam	ab2851	3 μg	Rabbit	Polyclonal
	DNMT1	ChIP, Co-IP	Abcam	ab13537	3 μg	Mouse	Monoclonal
	Rabbit IgG	ChIP, Co-IP	Abcam	ab37415	3 μg	Rabbit	Polyclonal
	Pol β	Immunoblotting	Abcam	ab175197	1:1000	Rabbit	Monoclonal
	DNMT3b	Immunoblotting	Santa Cruz Biotechnology	sc-376043	1:100	Mouse	Monoclonal
	DNMT1	Immunoblotting	Abcam	ab19905	1:1000	Rabbit	Polyclonal
**Secondary Antibodies**	m-IgGκ BP-FITC	Immunofluorescence	Santa Cruz Biotechnology	sc-516140	1:100	Mouse	Monoclonal
	Goat Anti-Mouse IgG H&L (Alexa Fluor® 594)	Immunofluorescence	Abcam	ab150116	1:1000	Goat	Polyclonal
	Goat Anti-Rabbit IgG H&L (Alexa Fluor® 488)	Immunofluorescence	Abcam	ab150077	1:1000	Goat	Polyclonal
	Goat Anti-Rabbit IgG H&L (HRP)	Immunoblotting	Abcam	ab6721	1:10000	Goat	Polyclonal
	Rabbit Anti-Mouse IgG H&L (HRP)	Immunoblotting	Abcam	ab6728	1:10000	Rabbit	Polyclonal

Table S2. Oligonucleotides sequences

Oligonucleotides	nt	Sequence (5'-3')[a]
**Upstream Strand**		
**U1**	38	GAATTCTTCCTCTTCCGTCTCTTTCCTTTTACGTCATC
**Downstream Strands**		
**D1**	37	pGGGGGCAGACTGGGTGGCCAATCCAGAGCCCCGAGAG
**D2**	37	p**F**GGGGGCAGACTGGGTGGCCAATCCAGAGCCCCGAGAG
**Template Strands**		
**T1**	76	CTCTCGGGGCTCTGGATTGGCCACCCAGTCTGCCCCC(**8-oxoG**) GATGACGTAAAAGGAAAGAGACGGAAGAGGAAGA ATTC
**T2**	76	CTCTCGGGGCTCTGGATTGGCCACCCAGTCTGCCCC(**5-mC**) (**8-oxoG**) GATGACGTAAAAGGAAAGAGACGGAAGAGGAAGA ATTC

[a] The damaged base is in boldface. F, tetrahydofuran; 8-oxoG, 8-hydroxyguanine; 5-mC, 5-methylcytosine.

Table S3. Primer sequences

Primers	nt	Sequence (5’-3’)
**<u>Bisulfate Sequencing</u>**		
Forward	36	GAGGCTAGAGGGCAGGCACTTTATGGCAAACTCAGG
Reverse	25	GTCCCCCGTCCAGGAAGTCTCAGCG
**<u>ChIP</u>**		
**BRCA1**		
Forward	21	GGCAGGCACTTTATGGCAAAC
Reverse	24	CAGTTATCTGAGAAACCCCACAGC
**β-actin**		
Forward	20	AGAGCTACGAGCTGCCTGAC
Reverse	20	AGCACTGTGTTGGCGTACAG

Supplementary Figure S1

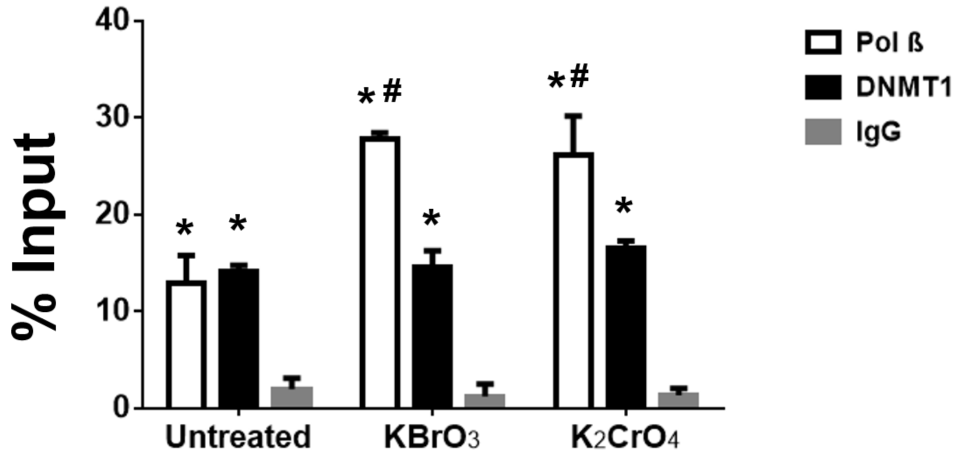

Supplementary Figure S1. The recruitment of pol β and DNMT1 to the promoter and transcribed regions of the BRCA1 gene. The recruitment of pol β and DNMT1 to the promoter and transcribed regions of the BRCA1 gene was detected by ChIP assay as described in the Materials and Methods. The quantification of the DNA amount that represents the amount of pol β and DNMT1 recruited to the regions of the BRCA1 gene in the untreated cells and cells treated with 5 mM bromate or 10 µM chromate was shown. The "% Input" was calculated using the equation: Input $\%=2^{-\Delta Ct\ [\text{normalized ChIP}]}\times 100$. It was obtained from three independent experiments and illustrated as mean ± S.D. Two-way ANOVA with Tukey's multiple comparison posttests was used to determine statistically significant differences. "*" denotes $P < 0.05$, compared to the IgG control, and "#" denotes $P <0.05$, compared with the untreated cells.

Supplementary Figure S2

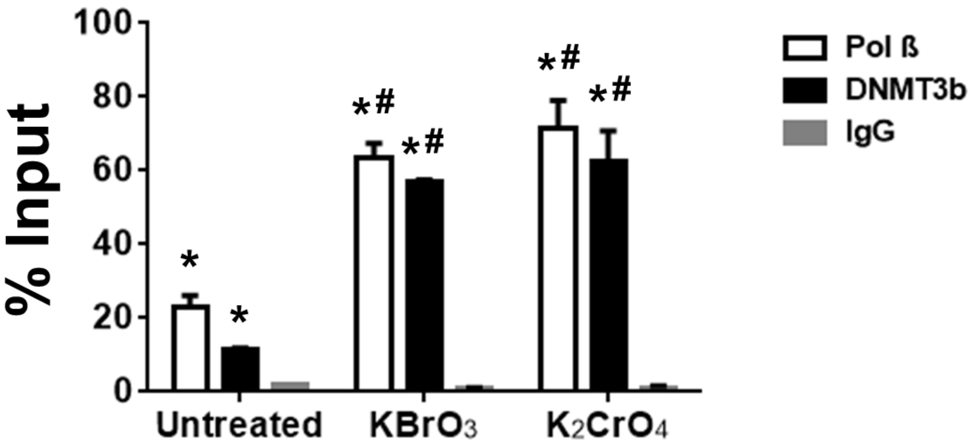

Supplementary Figure S2. The recruitment of pol β and DNMT3b to the β-actin gene. The recruitment of pol β and DNMT3b to the β-actin gene was detected by ChIP assay as described in the Materials and Methods. The quantification of the DNA amount that represents the amount of pol β and DNMT3b recruited to the regions of the β-actin gene in the untreated cells and cells treated with 5 mM bromate or 10 μM chromate was shown. The "% Input" was calculated using the equation: Input $\%=2^{-\Delta Ct\ [normalized\ ChIP]} \times 100$. It was obtained from three independent experiments and illustrated as mean ± S.D. Two-way ANOVA with Tukey's multiple comparison posttests was used to determine statistically significant differences. "*" denotes $P < 0.05$, compared to the IgG control, and "#" denotes $P < 0.05$, compared with the untreated cells.

Supplementary Figure S3

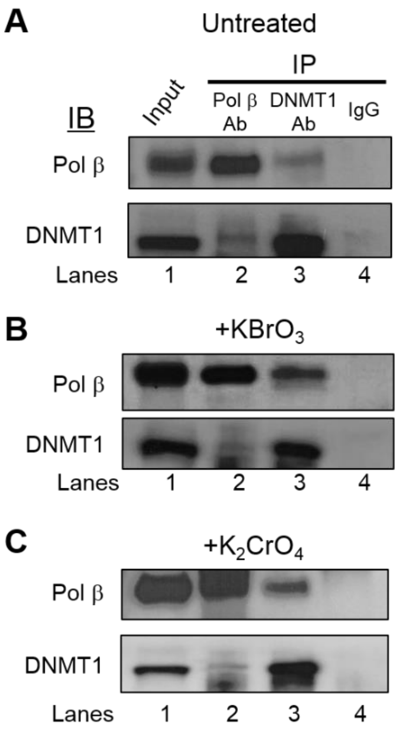

Supplementary Figure S3. Pol β interacts weakly with DNMT1 in cells. Co-IP and immunoblotting (IB) of pol β or DNMT1 in the extracts of untreated HEK293H cells (A) or HEK293 cells treated with 5 mM bromate (B) or 10 μM chromate (C) for 2 h. Cell lysates were subject to co-IP and IB for pol β and DNMT1, as described in the Materials and Methods. Lane 1 corresponds to cell lysates without treatment as an "Input" control. Lanes 2 and 3 correspond to cell lysates immunoprecipitated with an anti-pol β antibody and an anti-DNMT1 antibody, respectively. Lane 4 is the cell lysates immunoprecipitated with rabbit IgG alone. IP represents an immunoprecipitation antibody; IB indicates an immunoblotting antibody. All experiments were done in triplicate.
